# Enabling Psychiatrists to Explore the Full Potential of E-Health

**DOI:** 10.3389/fpsyt.2015.00177

**Published:** 2015-12-15

**Authors:** Melvyn W. B. Zhang, Roger C. M. Ho

**Affiliations:** ^1^National Addictions Management Service, Institute of Mental Health, Singapore; ^2^Department of Psychological Medicine, Yong Loo Lin School of Medicine, National University of Singapore, Singapore

**Keywords:** eHealth, mHealth, smartphone, psychiatry, mental disorders

## Definition of E-Health

The World Health Organization in its recent statement has highlighted the potential of the evolving field of E-Health (1). It defines E-health as the process in which health resources and health care are being communicated and transferred by electronic medium (1). It states explicitly that with the implementation of E-health, it is to be expected that there will be more efficient usage of healthcare resources in the future. Indeed, there has been major advances in the technological field over the past decade, especially so, with the introduction of Smartphone and their associated applications. Smartphones represent a new modality of technology that offers more than what conventional mobile technology could offer. Given that they are equipped with tremendous computing power, they now allow individuals and healthcare workers to access not only information but also work on the go. The authors acknowledge that E-health also encompasses other modalities of technologies, such as telephone-delivered therapy, virtual reality, and text messaging technology. In this manuscript, we will focus mainly on the smartphone aspect of E-health.

## Current State of Technological Adoption by Other Disciplines

The adoption of technology by healthcare professionals is not something new, as a previous systematic review (2) has highlighted that healthcare professionals are more receptive toward technology. The current technological advances implies that healthcare professionals are no longer confined to use individual workstations or computers, but they could have access to hospital records, laboratory results, and also latest updates in medical information at any location and at any time (2). This would indeed fulfill the World Health Organization’s vision of using technology to make the utilization of healthcare resources more efficient. Several disciplines have been early adopters of the evolving technologies, and they have utilized it for several key areas, such as in helping individuals manage their chronic diseases (3), in facilitating rehabilitation programs for patients (4), in helping clinicians with their medical diagnosis and lastly, helping to facilitate outreach efforts in developing countries (5). The utility of the current advances in technology would seemed unlimited, given that there have been other advances in this field, which include that of the development of robots, wearable computing, and wearable devices for monitoring and further advances in virtual reality technologies.

## Current State of Technological Adoption in Psychiatry with Regards to Smartphone Applications

Given the current advances in E-health and technologies, it is thus key in determining whether psychiatry has embraced these technologies developments and to what extent psychiatry has utilized these technological advances. A review of the existing published literature revealed that in psychiatry, these technologies advances have been utilized in several areas, such as the application of text-messaging services in reinforcing the medication compliance for schizophrenic patients (6), and also the usage of smartphone-based software to facilitate self-reporting of symptoms in schizophrenia patients (7). The current application of technologies is not limited to just patients with schizophrenia, but similar applications have been developed and implemented for patients with bipolar disorder, in helping them to monitor their mood states (8) as well as for assessment for depressive symptoms in patients (9). There has been previous research documenting the clinical efficacy of technologies in augmenting conventional psychotherapy in helping patients with substance-related disorders (9). Hence, it is beyond doubts that psychiatry, as a discipline, has tapped onto these advances in technologies and has embraced the technologies that E-health has introduced. In the recent edition of the British Journal of Psychiatry (10), an in-depth analysis has been given with regards to how psychiatrists could harness the best of this technological revolution. The authors of the paper that was published (10), pointed out the core issues of confidentiality and privacy issues, as well as the limitations in terms of the evidence base with regards to existing mobile phone and smartphone applications on the application store. The authors of the current paper do concur and acknowledge that these are crucial issues that need to be addressed first, prior to there being further utilization of technology for future advances in this field. It is the aim of the authors, to make use of this paper to address one of the main and core issues pertaining to the lack of evidence base with regards to existing published mobile applications and illustrate how psychiatrists could make use of simple methodologies to tap onto advances in this field, but yet ensure the evidence base of the products created, whether for education, research, or patient care. By doing so, it is thus hoped that psychiatrist would be able to explore the full potential of E-health.

## Methodologies for Psychiatrists to Tap onto Developments in This Evolving Arena

### Validation of Existing Applications Using Standardized Scales

Given that one of the core limitations identified pertaining to the implementation of E-health, especially so for smartphone applications for patient care, is the concern with regards to the safety of the application and the evidence base of the application, it is the aim of the authors to address this issue. One of the solutions might be getting psychiatrists to be involved in the identification of a collection of safe healthcare applications using systematic self-certification model. There have been previous studies conducted (11) that have proposed the application and the utilization of a systematic self-certification model for the peer review of applications. It is believed that such a methodology would be able to provide a systematic solution for healthcare workers in determining whether an application is safe and applicable for heir practice. One proposed solution involves the utilization of the self-certification model, which is based on the Health on the Net foundation (HON) guidelines. It is believed that this model would help in determining the reliability and credibility of the information presented on both medical and general health information websites. Table 1 illustrates in detail the aspects of this model.

**Table 1:** **Self-certification model**.

Description of criteria assessed by the HON guidelines
(a) Nature of information – it is recommended that all information included within the application should be attributed to an author. The qualifications of the author should be specified
(b) Purpose of the application – it is recommended that there should be a statement indicating the main purpose of the application
(c) Confidentiality – it is mandatory that the respective application documented how information is collected, and the confidentiality and privacy associated with the information collected
(d) Information – it is recommended that all medical information included should have a date of creation as well as a date documenting when the most recent update has been made
(e) Justification of claims – it is recommended that any information included pertaining to the benefits or the performance of any treatment should be supported by concrete scientific evidence
(f) Contact details – it is recommended that appropriate contact information should be included so that the respective developer or authors could be contacted
(g) Disclosures – all sources of funding should be disclosed
(h) Advertising policies – all advertisement included within the application should have a disclaimer indicating that they are advertisement and not scientific evidence

Another model, known as the Silberg scale (12), has also been used for the evaluation of the information quality of current applications (13, 14). More recently, a team of researchers in Australia has also proposed another scale, known as the mobile application rating scale (15). The scale has been developed after careful analysis of existing criteria for app evaluation, and the proposed scale has demonstrated good internal consistency and inter-rater reliability. Five core areas of an application could be evaluated by the scale, which ranged from that of engagement, functionality, esthetics, information quality, and a subjective quality scale (15). The development of such scales would greatly help psychiatrists in determining the evidence base pertaining to existing applications. Hence, it could facilitate a compilation of good evidence-based application that could then be recommended to patients.

Through the introduction of these scales, the authors hope that healthcare professionals could appreciate that there are methods that could be used to determine whether an application is evidence based or not.

### Enabling Psychiatrists to be Expert Developers

By providing psychiatrists with the above criteria, it is thus hoped that more psychiatrists could make use of the above-mentioned criteria to identify more evidenced-based applications. However, psychiatrists could tap upon the full potential of E-health if they could embrace methodologies of developing applications catered to the areas to which they could use smartphone applications in, for example, in education, research, and even in clinical care. Previous published research has encouraged clinicians to take upon more ownership of publishing application by means of educating them how best to make use of an Internet browser as well as a text editor to create applications (16). Zhang et al. (17, 18) in their recent published papers have further commented on how psychiatrists could become app developers, without the need to know programing skills. In their paper, online application builders were introduced to help facilitate this process. Apart from online application builders, they have also introduced responsive micro-blogging sites that could help create mobile friendly websites for instant reference on the go. In addition, they have highlighted how some of these builders could further facilitate the publication of applications onto the respective app stores, thus helping to minimize barriers with regards to the access of the applications.

## Conclusion

In conclusion, given the advances in technology in today’s day and age, it is essential for clinicians to tap upon technology and harness the full potential of today’s technologies. In order to empower psychiatrists, it is the perspective of the authors that psychiatrists could either make use of evidence-based methodologies to identify applications that are suitable for their usage, or empower themselves to be web-based and smartphone developers using simple methodologies shared previously. Given the simplicity and the low-cost involved using the methods introduced (17, 18), it is hoped that psychiatrists would be willing to put their ideas into action and help in the formulation of more evidence-based applications.

## Conflict of Interest Statement

The authors declare that the research was conducted in the absence of any commercial or financial relationships that could be construed as a potential conflict of interest. The reviewer Jérôme Favrod and handling Editor Stéphane Bouchard declared a current collaboration (working on the current Research Topic). The handling Editor states that the process nevertheless met the standards of a fair and objective review.
